# Draft genome sequence of type strain *Streptomyces brasiliscabiei* IBSBF 2867^T^

**DOI:** 10.1128/MRA.00370-23

**Published:** 2023-08-02

**Authors:** Daniele Bussioli Alves Corrêa, Lucas Vitor, Danilo Trabuco do Amaral, Mariana Ferreira-Tonin, Suzete Aparecida Lanza Destéfano

**Affiliations:** 1 Laboratório de Bacteriologia Vegetal, Centro Avançado de Pesquisa e Desenvolvimento em Sanidade Agropecuária (CAPSA), Instituto Biológico, Campinas, São Paulo, Brazil; 2 Departamento de Biologia, Centro de Ciências Humanas e Biológicas, Universidade Federal de São Carlos (UFSCar), Sorocaba, São Paulo, Brazil; 3 Programa de Pós Graduação em Biologia Comparada, Faculdade de Filosofia, Ciências e Letras de Ribeirão Preto, Universidade de São Paulo (USP), Ribeirão Preto, São Paulo, Brazil; The University of Arizona, Tucson, Arizona, USA

**Keywords:** potato scab, phytopathogenic *Streptomyces*

## Abstract

Here, we report the draft genome sequence of *Streptomyces* IBSBF 2867^T^, associated with potato scab in Brazil. Genome analysis using the antiSMASH bioinformatics tool showed the presence of phytopathogenic biosynthetic pathways.

## ANNOUNCEMENT

Potato scab, caused by phytopathogenic *Streptomyces*, is a complex disease that affects the crop with widespread occurrence in the main producing regions of Brazil and the world (1, 2). The symptoms are characterized by lesions on the tuber surface, which decrease its commercial value or even prevent commercialization, both for consumption and to serve as seeds (3, 4).

Potato tubers exhibiting scab lesions, from production area in Santa Catarina State, Brazil, were disinfected with 1.5% NaOCl/1 min and rinsed with sterile distilled water. After cutting, the scab lesions were placed in a tube with sterile water, incubated at 55°C/30 min, and then macerated in a microscope slide with sterile water. A drop of this suspension was streaked onto water agar medium, and the plates were incubated at 28°C/10 d. Characteristic colonies of *Streptomyces* were picked, transferred to ISP 2 medium at 28°C/14 d (5), and subsequently identified as *S. brasiliscabiei* IBSBF 2867^T^. The type strain showed morphological, physiological, and biochemical characteristics very similar to *S. scabiei*, bacterial species widely distributed in the world, as well as the main pathogenicity factors: taxtomin, *nec* 1, and tomatinase, but with different genomic features (1).

DNA extraction of *S. brasiliscabiei* IBSBF 2867^T^ was performed according to Corrêa et al. (6), and the genome sequencing was carried out by Biotecnologia Pesquisa e Inovacão, Botucatu, Brazil, using the Illumina HiSeq 2500 next-generation sequencing platform with a 2 × 100 bp paired-end protocol. The sequencing library was prepared using the TruSeq DNA library preparation kit with the standard Illumina DNA protocol. The raw reads were quality trimmed (including adapters removal) using Seqyclean software and *de novo* assembled using SPAdes 3.14.1 (7) in ‘‘isolate’’ mode. The SSPACE_basicv2.pl script (8) was used to improve the scaffold pre-assembled contigs using the paired-end library, and the quality of the genome assembly was assessed using QUAST (9). The draft genome, genes, and proteins were annotated with the DFAST web-based genome annotation tool (10). Default parameters were used for all software unless otherwise specified.

The whole-genome sequencing resulted in, approximately, 2.1 Gb raw reads. *De novo* genome was assembled into 1,319 scaffolds (N_50_, 40,518 bp), comprising a total of 10,846,379 bp with a GC content average of 71.3%. The draft genome annotation predicted a total of 9,179 putative genes, 3 rRNAs, 89 tRNAs, and 1 CRISPR annotated. The highest percentage of genes is involved in catalytic activity (~15%) and in metabolic processes within the category of biological process.

Analysis using antiSMASH 6.0 online tool showed 42 biosynthetic clusters associated with different secondary metabolites were also identified. Two predicted pathways showed high homology to biosynthetic plant pathology gene clusters: *thaxtomin D/thaxtomin A/thaxtomin C/thaxtomin B* gene clusters showed 83% similarity with *Streptomyces scabiei* 87.22, and the *concanamicin A* gene cluster showed 82% similarity with *Streptomyces neyagawaensis*.

## Data Availability

The whole-genome sequence has been deposited at DDBJ/ENA/GenBank under the accession number PRJNA632639, for the assembly JABRXD000000000.1, and reads in SRA SRX8353089. The version described in this paper is the first version.
